# The effect of outdoor activities on the medical expenditure of older people: multiple chain mediating effects of health benefits

**DOI:** 10.1186/s12889-024-18719-z

**Published:** 2024-05-03

**Authors:** Ge Zhu

**Affiliations:** School of Economics, Trade and Management, Gansu University of Chinese Medicine, Lanzhou, Gansu, China

**Keywords:** Older people, Outdoor activities, Medical expenditures, Multiple chain mediation effects, Health benefits

## Abstract

**Background:**

With the global aging population, attention to the health and medical issues of older adults is increasing. By analyzing the relationship between older people's participation in outdoor activities and medical expenditure, this study aims to provide a scientific basis for improving their quality of life and reducing the medical burden.

**Methods:**

Data on outdoor activity participation, medical expenditures, and relevant variables were collected through questionnaires and databases. A multi-chain mediation effect model was established to analyze the impact of outdoor activities on the medical expenditure of older people, considering mediation effects and heterogeneity.

**Results:**

Results revealed that increased participation in outdoor activities among older adults correlated with lower medical expenditures. Outdoor activities positively influenced their health by improving mental health, cognition, eating habits, and activities of daily living, resulting in reduced medical expenditures. Robustness tests confirmed the consistent effect of outdoor activities on older people's medical expenditure.

**Conclusion:**

These findings contribute to understanding the relationship between outdoor activities, health, and medical expenditure in older people, guiding policy formulation and interventions. Encouraging and supporting older adults in outdoor activities can enhance their quality of life and alleviate medical resource strain. The study's conclusions can also inform health promotion measures for other populations and serve as a basis for future research in this area.

## Introduction

China's aging population presents challenges to medical, necessitating a focus on the health of middle-aged and older adults’ individuals. The "Healthy China 2023" Planning Outline emphasizes the importance of managing common and chronic diseases among older people. Participating in outdoor activities for older people can reduce the prevalence of diseases, improve quality of life, and control medical expenses. This issue is not unique to China but is a global concern. Studying the impact of outdoor activities on medical spending reveals their benefits for physical, social, and psychological well-being, offering a scientific basis for improving the health and quality of life for older people. Understanding the relationship between outdoor activities and medical expenses enables policymakers to allocate resources effectively and develop health policies. While this study focuses on older people, its findings have implications for all age groups, highlighting the research value of understanding outdoor activities' impact on medical spending.

Despite the importance of studying the impact of outdoor activities on medical spending, there are several challenges in conducting such research. First, accurately measuring the health benefits attributed to outdoor activities is challenging, as individuals engage in activities of varying intensity each day. Second, this research spans multiple disciplines, necessitating interdisciplinary knowledge and expertise, which can limit the scope and depth of the study. Third, investigating the causal relationship between outdoor activities and medical spending requires long-term follow-up studies to address confounding factors. Factors like individual health status and lifestyle habits complicate research design and data analysis. To mitigate these challenges, this study focuses on older people population and utilizes a large database with extensive samples to compensate for the inability to track participants over a long period. A multi-chain mediating effect model is employed, using mediating variables such as mental health, cognitive ability, eating habits, daily activities, and general health status to examine the specific impact of outdoor activities on medical expenditure while addressing potential interference factors.

**Fig. 1:**
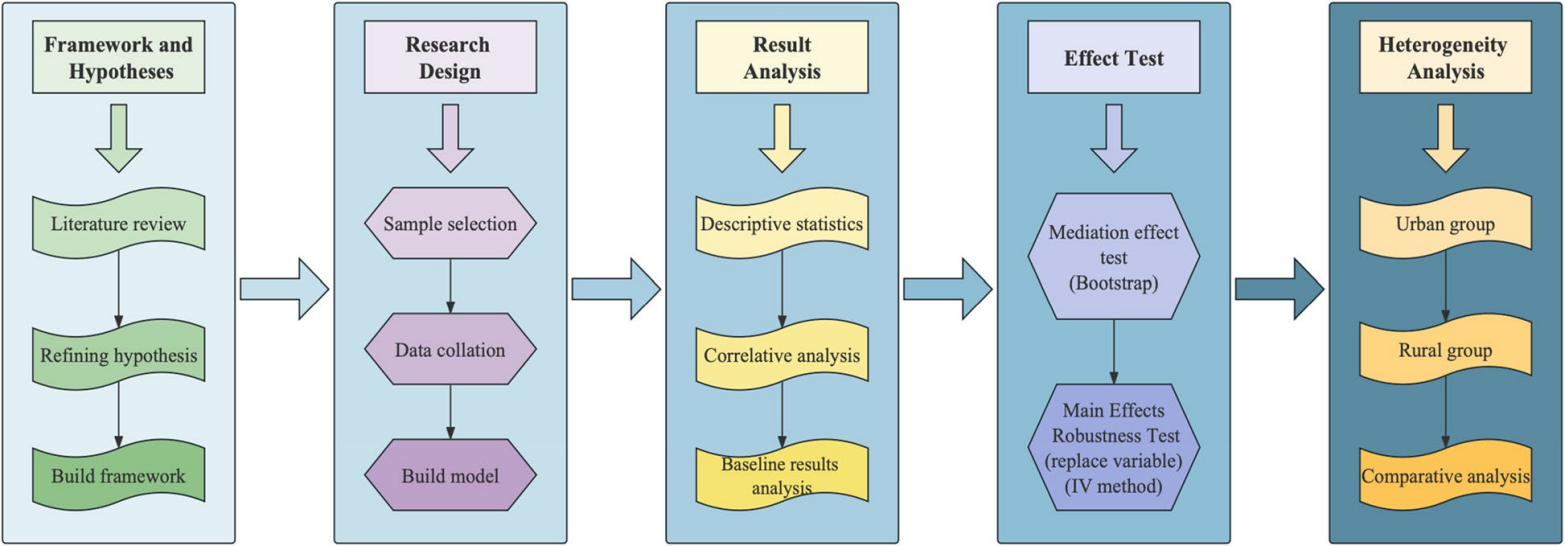
Research structure and process. Note: This figure shows the main research framework of our article, which we can split into a horizontal research process and a vertical research process. The horizontal research process mainly includes the process from the establishment of the theoretical framework and assumptions to the heterogeneity analysis, and the longitudinal research process subdivides each horizontal research, which is a shallow and deep process. As can be seen from the figure, the core of the study lies in the analysis of the main results and the verification of the robustness of the results, and on this basis, group regression (heterogeneity analysis) was additionally explored

This study will be divided into the following sections to discuss. First, we will review the relevant literature, including exploring the possible health benefits of outdoor activity among older adults and outlining the positive impact of these health benefits on medical spending. Next, we will introduce the research design of this study, including data collection and model building. Then, we will present and discuss our findings, including detailed outdoor activities, the impact paths, and mediation effects between various mediator variables and medical expenditure. At the same time, we further classified older people group and explored the different performances of urban and rural older adults’ groups in the impact mechanism of outdoor activities on medical expenditure. To verify the robustness of the above-mentioned mediation effect, we changed the Variables and methods for using instrumental variables. Finally, we summarize the main findings of the study. Meanwhile, Fig. 1 shows the detailed research process of this study.

## Literature review and research hypothesis

### Health benefits of outdoor activities for older adults

Outdoor activities combine outdoor time and physical activity, offering a balanced approach to health benefits. Unlike indoor sports, outdoor sports provide more space, connecting people with nature and each other. While we recognize the benefits of physical activity, we often overlook the impact of indoor versus outdoor environments on these benefits. Engaging in physical activity outdoors has additional advantages, including increased energy, active engagement, and reduced tension, confusion, anger, and depression compared to indoor exercise. This is particularly important for older people, who often have more flexibility in their daily routines. To understand the impact of outdoor activities on health, we must consider both outdoor time and physical activity, as they influence mental health, cognitive abilities, eating habits, and daily routines [1].

#### Outdoor activities affect mental health

Social support and relationships are crucial for mental health. Strong social connections in older adults offer emotional, informational, and substantive support, reducing stress and aiding in coping with psychological difficulties. Research indicates that social support is linked to decreased symptoms of depression and anxiety, increased well-being, and greater life satisfaction [2]. On the other hand, social isolation and loneliness heighten the risk of mental health issues, including depression, anxiety, and suicidality. Additionally, the natural environment plays a significant role in mental health. Spending time in parks, forests, and beaches can alleviate stress, improve mood, and enhance attention stability. Green landscapes and natural elements help alleviate mental fatigue and enhance psychological well-being. Furthermore [3], physical activity has a positive impact on mental health. Engaging in moderate physical activity releases chemicals such as endorphins and dopamine, promoting positive emotions and a sense of well-being. Regular physical activity reduces symptoms of depression and anxiety while boosting self-esteem and self-efficacy. Therefore, we get the following assumptions:

- H1a: Outdoor activities have a positive effect on the improvement of the mental health of older people.

#### Outdoor activities affect cognitive ability

Maintaining positive social connections benefits cognitive performance in older adults by stimulating cognitive function. Social activities, communication, and group participation enhance cognitive flexibility, memory, and attention. Social support reduces cognitive decline and delays dementia. Close contact with loved ones and community involvement combats social isolation and preserve cognitive function. Exposure to natural environments improves cognitive performance by providing sensory and cognitive stimulation [4]. Nature walks and landscape viewing enhance working memory, processing speed, and creative thinking in older people. The tranquility and beauty of nature relieve cognitive fatigue and support recovery. Gardening and outdoor activities offer cognitive benefits. Moderate physical activity, such as walking, dancing, and swimming, enhances brain function by increasing blood circulation and oxygen supply. These aerobic exercises improve attention, memory, and executive function in older adults. Physical activity also promotes neurogenesis, synaptic connections, and brain health, reducing the risk of cognitive decline and dementia [5]. Therefore, we get the second hypothesis:

- H1b: Outdoor activities have a positive effect on the improvement of cognitive ability in older people.

#### Outdoor activities affect eating habits

Social distancing and eating habits are intertwined. Positive social connections lead to healthier eating choices in older adults. Interacting with others and sharing meals support nutritious dining experiences [6]. Dining with companions enhances appetite and promotes food intake. Exposure to the natural environment is linked to better eating habits. Natural environments provide fresh ingredients, organic food, and abundant plant-based resources. Studies show that exposure to nature encourages preference for nutritious foods such as fruits, vegetables, and whole grains [7]. Additionally, natural environments have a calming effect, reducing emotional and binge eating tendencies. Moderate physical activity positively affects the eating habits of older adults. It boosts metabolism, increases appreciation for healthy foods, and promotes a balanced diet. Physically active older adults prioritize vegetables, fruits, lean meats, and whole grains. Physical activity also improves digestion, appetite, and food absorption while reducing emotional eating and stress-related poor eating habits [8]. Therefore, we get the third hypothesis:

- H1c: Outdoor activities have a positive impact on the development of good eating habits in older people.

#### Outdoor activities affect daily activities

Positive social relationships and support enhance daily activities in older adults by providing emotional and practical assistance, boosting self-confidence and motivation, and improving the development and maintenance of daily living skills [9]. Social interaction reduces loneliness and depression, enhances self-management, and adds meaning to their lives [10]. A favorable natural environment has a positive impact on the daily activities of older people [11]. Exposure to nature promotes relaxation, reduces stress and fatigue, and improves mental health. Natural spaces like parks, gardens, and walking paths encourage physical activity, enhancing muscle strength, flexibility, and balance. Regular physical activity is crucial for older adults, improving muscle strength, flexibility, and the ability to perform daily tasks like walking and climbing stairs. It also enhances cardiovascular fitness, increases energy and endurance, reduces fatigue, and improves overall physical function. We get the fourth hypothesis:

- H1d: Outdoor activities have a positive effect on the improvement of the daily activities of older people.

### Health factors affecting medical expenditure of older people

The literature review highlighted that outdoor activities have positive effects on the mental health, cognitive ability, eating habits, and daily activities of older people. However, no established study has measured the specific impact of outdoor activities on physical and mental health. To assess the health benefits, we propose using the medical expenditure of older people as an indicator, as we believe that outdoor activities directly influence these expenses and, in turn, the overall health benefits. The logic is that the unhealthy state of older adults will be accurately reflected in medical expenditures, and when this state is improved, the reduction in medical expenditures can accurately reflect the overall health benefits of older adults. Overall health benefits are relative. When older adults improve their health through outdoor activities. Seniors who participate in outdoor activities enjoy more health benefits than those who do not participate in outdoor activities, which is reflected in reduced medical expenditures [12]. Therefore, we get the fifth hypothesis:

- H6: Outdoor activities will reduce the medical expenditure of older people.

#### Mental health affects medical expenditure

Depression and anxiety are associated with chronic diseases [13], particularly in older adults who are more prone to conditions such as cardiovascular disease, diabetes, and chronic respiratory diseases [14]. These mental health issues can affect the management and treatment of chronic diseases, leading to increased medical costs. Older adults with depression and anxiety may neglect their medical needs and delay seeking medical assistance, which can worsen their conditions and result in higher medical spending [15]. Furthermore, mental health problems in older adults contribute to increased utilization of medical services due to physical discomfort, illness, and functional impairment [16]. They also contribute to medical errors, frequent doctor visits, longer hospital stays, and higher medical expenses. Treating mental health problems in older adults often involves long-term medication, adding to the financial burden of medical expenditures. Thus, we get the sixth hypothesis:

- H2a: The improvement of mental health can reduce the medical expenditure of older people.

#### Cognitive ability affects medical expenditure

Lower cognitive ability in older adults may hinder their management of chronic diseases, including medication adherence, diet control, and medical appointments, resulting in poor disease control and higher medical expenses [17]. Additionally, reduced cognitive abilities can impede medical decision-making and communication [18], leading to unnecessary tests, repeated doctor visits, and excessive medical bills. Furthermore, lower cognitive abilities increase the need for care and supervision, such as long-term care and nursing homes, thereby raising medical costs [19]. Lastly, cognitive decline in older adults elevates the risk of unintentional injuries and accidents, necessitating emergency treatment and rehabilitation care, which further contributes to medical expenses [20]. Thus, we get the seventh hypothesis:

- H2b: The improvement of cognitive ability can reduce the medical expenditure of older adults.

#### Eating habits affect medical expenditure

Poor dietary habits in older people, including alcoholism, smoking, irregular diet, intake of junk food, unbalanced nutritional intake, etc. which increase the risk of chronic diseases, requiring long-term medical management and incurring high expenses. Inadequate nutrient intake due to reduced physical function and appetite can cause malnutrition, leading to diminished immunity, muscle atrophy [21], osteoporosis, and increased medical costs. Unhealthy eating habits can also contribute to digestive system problems like indigestion, gastric ulcers, and constipation, necessitating medical intervention and escalating medical spending [22]. Additionally, these habits can raise the risk of nutrition-related conditions such as obesity, hyperlipidemia, and gout, demanding extended medical care and amplifying expenses. Poor eating habits in older people may also increase the likelihood of food safety issues like food poisoning and bacterial infections, requiring emergency room visits, medical treatment, and further medical expenditure. Thus, we get the eighth hypothesis:

- H2c: Improvement of eating habits will reduce the medical expenditure of older adults.

#### Ability of daily activities affects medical expenditure

Limited daily activities in older adults may heighten the risk of accidents, such as falls, slips, and fractures, resulting in increased medical costs for emergency treatment and rehabilitation [23]. Impaired daily activities can also impact their ability to manage chronic diseases, leading to worsened symptoms, frequent medical visits, and higher medical expenses [24]. Additionally, reduced daily activities may necessitate long-term care, including assistance with daily living tasks, which further contributes to medical expenditures. Furthermore, older adults with diminished daily activities may require additional rehabilitation services, such as physical and occupational therapy, and the use of rehabilitation equipment, increasing medical spending [25]. Thus, we get the ninth hypothesis:

- H2d: Improved ability to perform daily activities reduces medical expenditure in older adults.

### Other paths

Examination of the literature on mental health, cognitive ability, eating habits, activities of daily living, and medical costs of older adults reveals a high degree of association between medical costs and the health benefits of outdoor activities in health economics studies, and that these health indicators are It is a form of health status. Thus, we get the remaining assumptions:

- H3a: The improvement of mental health will lead to the improvement of the general health status of older adults;
- H3b: Improvement in cognitive ability will lead to improvement in the general health status of older adults;
- H3c: Improvement in eating habits will lead to an improvement in the overall health status of older adults;
- H3d: Improvements in activities of daily living led to improvements in the general health status of older adults;
- H4: Outdoor interaction will have a positive impact on the general health status of older adults;
- H5: The improvement of overall health status will reduce the medical expenditure of older adults.

**Fig. 2:**
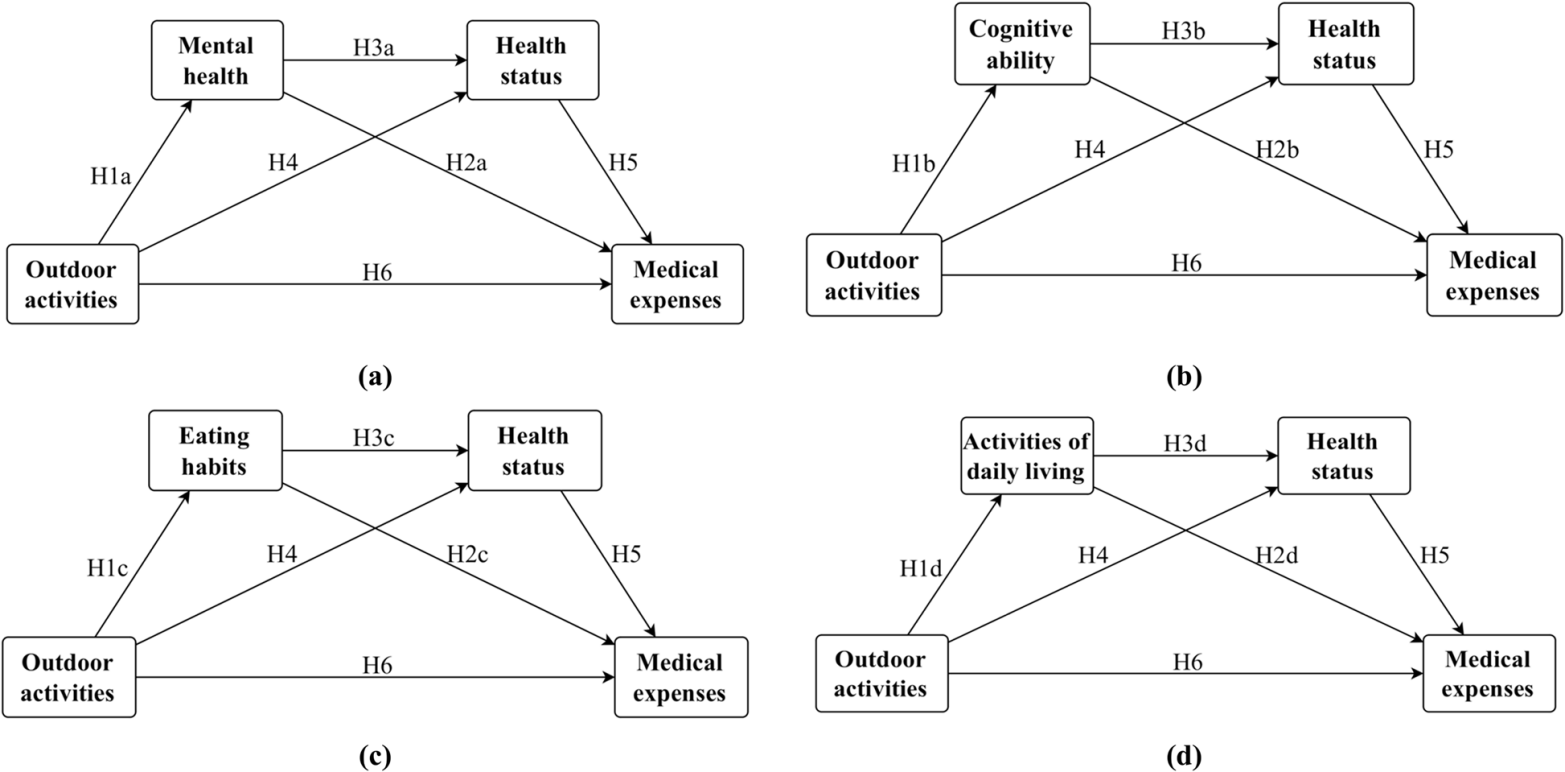
Transduction pathways and research framework. Note: The figure shows a clear path of influence based on various hypotheses obtained from the literature review analysis, in which (**a**) represents the establishment of a double-chain mediation effect under the mediation variables of the mental health and overall health of older people; **b** represents older people’s the cognitive ability and health status are the mediating variables to establish a double-chain mediation effect; **c** indicates the establishment of a double-chain mediation effect when older people’s eating habits and health status are the mediating variables; **d** indicates that older people’s daily Living ability and health status are mediating variables to establish a double-chain mediating effect

As shown in Fig. 2, we express all the research paths and research hypotheses in the form of pictures. From the figure, it is not difficult to find that the research mainly revolves around 15 hypotheses. It includes 3 important hypotheses (H4, H5 and H6) and 12 secondary hypotheses (H1a, H1b, H1c, H1d, H2a, H2b, H2c, H2d, H3a, H3b, H3c and H3d). At the same time, these 12 secondary hypotheses are mainly based on the two important assumptions mentioned above, namely H4 and H6. Next, we will sequentially verify and analyze the above assumptions according to the path and framework shown in Fig. 2.

## Research design

To further test the direct and indirect relationship between outdoor activities and medical expenditures of older people, we selected the relevant survey data of older people in China, selected relevant indicators, and built an empirical model to test the relationship between outdoor activities and medical expenditures. Whether the selected mediator variables play a significant mediating role in the way outdoor activities affect the medical expenditure of older people. Table 1 shows the main variables of this study and their definitions.

**Table 1:** Selection and definition of variables

Variable type	Variable name	Variable code	Variable definitions
Explained variable	Medical expenditure	ME	Inpatient expenses + outpatient expenses, this is a continuous variable
Explanatory variable	Outdoor activities	OA	Frequency of older people participating in outdoor low-intensity Physical activities performed outdoors such as Tai Chi and square dancing, this is a categorical variable. Specifically, almost never participating in outdoor activities was coded as 1, frequency as a year was coded as 2, frequency as a month was coded as 3, frequency as a week was coded as 4, and participating almost every day was coded as 5
Mediator variables	Health status	HS	Older people's self-perceived comprehensive health status, this is a categorical variable. Specifically, very bad is marked as 1, bad as 2, average as 3, good as 4, and very good as 5
	Mental health	MH	100—Depression level (percentile depression scale), this is a continuous variable. The score is calculated based on responses to the depression scale section of the questionnaire
	Cognitive ability	CA	Awareness and understanding of things, this is a continuous variable. Calculated based on the cognitive function scale of the questionnaire, the total score is 100 points
	Eating habits	EH	Intake frequency of fresh vegetables and fruits in daily life habits and Intake frequency of alcohol and tobacco in daily life habits, this is a categorical variable. Having more good habits and fewer bad habits will be defined as closer to 5, and vice versa will be defined as closer to 1
	Activities of daily living	ADL	The necessary activities that older people perform every day to meet the needs of daily life. this is a continuous variable. Calculations were based on the daily activity ability scale of the questionnaire
Control variables	Place of residence	PR	The place where you live in your daily life, city (marked as 1), town (marked as 2) or village (marked as 3), this is a categorical variable
	Age	Age	The number of years older people lived when surveyed, this is a continuous variable
	Family size	Size	Number of people living with older people (the older people themselves are counted), this is a continuous variable
	GDP per capita	GDP	Total GDP/total population, statistics by province, this is a continuous variable

Among them, we selected five variables as intermediary variables. According to the theoretical framework, we took MH, CA, EH, and ADL as parallel variables, which would affect HS. When we study any one parallel variable, other parallel variables are taken as control variables. We will show the calculation details and references about MH, CA, EH, and ADL in the appendix section

### Sample selection and data sources

We primarily utilized data from the China Elderly Health Survey (CLHLS), an official database conducted by the Center for Healthy Aging and Development of Peking University and the National Development Research Institute of China. Supplementary research was also conducted using data from the China Statistical Yearbook. Specifically, we focused on the "2018" dataset within the CLHLS, which surveyed older adults’ individuals across different regions of China from 2017 to 2019, capturing their individual, family, and social characteristics. To ensure accuracy and maximize the sample size, we implemented the following measures to remove older adults’ samples from the dataset: excluding those with missing core variables, replacing missing control variables with the overall sample average, resulting in a total of 15,874 relevant older adults’ data points (Eliminate 89 failed samples, accounting for 0.56% of the original samples). Among them, female account for 56.38%. All data used in this study originate from this dataset, and Stata17.0 software was employed for regression analysis and model testing [26].

### Model establishment

To test the hypotheses, this paper establishes a relationship model examining the impact of outdoor activities on health status, mental health, cognitive ability, eating habits, activities of daily living (ADL), and medical expenditure. Using Baron and Kenny's three-step test method (1986) [27], we incorporate mediator variables into the model and analyze the changes in regression results to verify the mediation effect. The intermediary variables in this study include health status, mental health, cognitive ability, eating habits, and daily activities. We hypothesize a chain mediating effect, specifically the influence of mental health, cognitive ability, eating habits, and daily activities on health status. The following are the modeling steps for this chained mediation effect:

First, it is necessary to construct a total effect model of the impact of outdoor activities on medical expenditure:

1 $${ME}_{it}={\alpha }_{10}+{\alpha }_{11}{OA}_{it}+{\alpha }_{12}{PM}_{1it}+{\alpha }_{13}{PM}_{2it}+{\alpha }_{14}{PM}_{3it}+{\alpha }_{15}{PM}_{4it}+{\alpha }_{16}{PR}_{it}+{\alpha }_{17}{Age}_{it}+{\alpha }_{18}{Size}_{it}+{\alpha }_{19}{GDP}_{it}+{\varepsilon }_{1it}$$

Among them, ${ME}_{it}$ is the medical expenditure of older people i in year t, ${\alpha }_{10}$ is the constant item, ${\varepsilon }_{1}$ is the residual item, ${\alpha }_{11}$ represents the impact coefficient of outdoor activity frequency on medical expenditure, and ${\alpha }_{12}-{\alpha }_{19}$ represents the impact of each control variable on medical expenditure coefficient. Here, model (1) is the total effect of outdoor activities on medical expenditure under the framework of health status and another variable (mental health, cognitive ability, eating habits, daily activities) as mediating variables, so we will divide the three mediator variables to be studied other than the mediator variables are used as control variables and expressed as parallel mediator variables (PM1, PM2, PM3). If ${\alpha }_{11}$ is significantly negative, test hypothesis H6, that is, the increase in the frequency of outdoor activities of older people can help reduce medical expenditure.

Secondly, the models of the influence of outdoor activity frequency on mental health, cognitive ability, eating habits, and daily activities were constructed respectively:

2 $${MH}_{it}={\alpha }_{20}+{\alpha }_{21}{OA}_{it}+{\alpha }_{22}{CA}_{it}+{\alpha }_{23}{EH}_{it}+{\alpha }_{24}{ADL}_{it}+{\alpha }_{25}{PR}_{it}+{\alpha }_{26}{Age}_{it}+{\alpha }_{27}{Size}_{it}+{\alpha }_{28}{GDp}_{it}+{\varepsilon }_{2it}$$

3 $${CA}_{it}={\alpha }_{30}+{\alpha }_{31}{OA}_{it}+{\alpha }_{32}{MH}_{it}+{\alpha }_{33}{EH}_{it}+{\alpha }_{34}{ADL}_{it}+{\alpha }_{35}{PR}_{it}+{\alpha }_{36}{Age}_{it}+{\alpha }_{37}{Size}_{it}+{\alpha }_{38}{GDp}_{it}+{\varepsilon }_{3it}$$

4 $${EH}_{it}={\alpha }_{40}+{\alpha }_{41}{OA}_{it}+{\alpha }_{42}{CA}_{it}+{\alpha }_{43}{MH}_{it}+{\alpha }_{44}{ADL}_{it}+{\alpha }_{45}{PR}_{it}+{\alpha }_{46}{Age}_{it}+{\alpha }_{47}{Size}_{it}+{\alpha }_{48}{GDp}_{it}+{\varepsilon }_{4it}$$

5 $${ADL}_{it}={\alpha }_{50}+{\alpha }_{51}{OA}_{it}+{\alpha }_{52}{CA}_{it}+{\alpha }_{53}{EH}_{it}+{\alpha }_{54}{MH}_{it}+{\alpha }_{55}{PR}_{it}+{\alpha }_{56}{Age}_{it}+{\alpha }_{57}{Size}_{it}+{\alpha }_{58}{GDp}_{it}+{\varepsilon }_{5it}$$

Among them, ${MH}_{it}$, ${CA}_{it}$, ${EH}_{it}$, ${ADL}_{it}$ are respectively the mental health, cognitive ability, eating habits and daily activity ability of older people i in t years, and ${\alpha }_{20}$, ${\alpha }_{30}$, ${\alpha }_{40}$, ${\alpha }_{50}$ are Constant items, ${\varepsilon }_{2}$, ${\varepsilon }_{3}$, ${\varepsilon }_{4}$, ${\varepsilon }_{5}$ are residual items, ${\alpha }_{21}$, ${\alpha }_{31}$, ${\alpha }_{41}$, ${\alpha }_{51}$ represent the influence coefficient of outdoor activities on the dependent variable, ${\alpha }_{22}-{\alpha }_{28}$, ${\alpha }_{32}-{\alpha }_{38}$, ${\alpha }_{42}-{\alpha }_{48}$, ${\alpha }_{52}-{\alpha }_{58}$ 8 represent the control The coefficient of influence of the variable on the dependent variable. If ${\alpha }_{21}$, ${\alpha }_{31}$, ${\alpha }_{41}$, ${\alpha }_{51}$ are all significantly positive, the hypotheses H1a, H1b, H1c, and H1d will be verified, that is, increasing the frequency of outdoor activities will help improve the mental health, cognitive ability, eating habits, and daily activities of older people.

Secondly, according to previous studies, we judge that there may be a chain intermediary effect of "OA → MH (CA, EH, ADL) → HS → ME", so we should build a corresponding model for this:

6 $${HS}_{it}={\alpha }_{60}+{\alpha }_{61}{OA}_{it}+{\alpha }_{62}{ME}_{it}+{\alpha }_{63}{CA}_{it}+{\alpha }_{64}{EH}_{it}+{\alpha }_{65}{ADL}_{it}+{\alpha }_{66}{PR}_{it}+{\alpha }_{67}{Age}_{it}+{\alpha }_{68}{Size}_{it}+{\alpha }_{69}{GDp}_{it}+{\varepsilon }_{6it}$$

Finally, we construct a direct effect model of the impact of outdoor activity frequency on medical expenditure:

7 $${ME}_{it}={\alpha }_{70}+{\alpha }_{71}{OA}_{it}+{\alpha }_{72}{ME}_{it}+{\alpha }_{73}{CA}_{it}+{\alpha }_{74}{EH}_{it}+{\alpha }_{75}{ADL}_{it}+{\alpha }_{76}{HS}_{it}+{\alpha }_{77}{PR}_{it}+{\alpha }_{78}{Age}_{it}+{\alpha }_{79}{Size}_{it}+{\alpha }_{710}{GDp}_{it}+{\varepsilon }_{7it}$$

Among them, ${ME}_{it}$ is the medical expenditure of older people i in year t, ${\alpha }_{70}$ is the constant item, ${\varepsilon }_{7}$ is the residual item, ${\alpha }_{71}$, ${\alpha }_{72}$, ${\alpha }_{73}$, ${\alpha }_{74}$, ${\alpha }_{75}$, ${\alpha }_{76}$ represent the frequency of outdoor activities, mental health, cognitive ability, The influence coefficients of eating habits, daily activities and health status on medical expenditures, if these coefficients are significantly negative, then verify the hypotheses H2a, H2b, H2c, H2d, H5, that is, outdoor activities, mental health, cognitive ability, eating habits, Activities of daily living and health status are conducive to reducing medical expenditures in older people. ${\alpha }_{77}-{\alpha }_{710}$ represents the influence coefficient of each control variable on the dependent variable.

## Results analysis

### Descriptive statistics

Table 2 displays descriptive statistics. The per capita medical expenditure of older people is 6434.86 yuan annually, with a large standard deviation, suggesting a wide variation in medical expenses among older people, The majority of older people do not incur any medical costs throughout the year. As per data from the Ministry of Social Security in 2022, the average monthly pension is approximately 3,500 yuan. For those whose medical expenses align with this average, two months' pension can cover the entire year's medical costs, alleviating concerns about payment. However, older adults’ individuals with high medical expenditures may struggle to fully cover their expenses with the pension alone, necessitating support from both family and social insurance. Improving older adults’ health and reducing medical costs benefits all parties involved. Older adults typically engage in outdoor activities once a month, indicating that overall health issues in an aging society are not highly prevalent. Cognitive ability among older people tends to be higher on average, indicating that dementia is relatively uncommon. However, their mental health, eating habits, and daily life abilities fall below the average line, suggesting that society and families have overlooked the psychological state and daily care of older people.

**Table 2:** Descriptive statistics

Variables	Code	Obs	Mean	Std	Min	Max
Medical expenditure	ME	15,874	6434.86	28,815.65	0	389,174
Outdoor activities	OA	15,874	3.51	1.56	1	5
Health status	HS	15,874	3.39	0.87	1	5
Mental health	MH	15,874	56.71	12.03	4	90
Cognitive ability	CA	15,874	81.83	21.52	23.33	100
Eating habits	EH	15,874	2.95	1.25	1	5
Activities of daily living	ADL	15,874	50.82	19.92	0	78
Place of residence	PR	15,874	2.22	0.79	1	3
Age	Age	15,874	85.46	11.69	60	117
Family size	Size	15,874	2.97	1.94	1	22
GDP per capita	GDP	15,874	72,769.91	32,552.11	36,441	161,776

We have positively processed all the above variables. The larger the value of the mental health variable, the healthier older people are; the greater the cognitive ability, the stronger the cognitive ability of older people; eating habits are categorical variables. The larger the value, the older people consume more fresh fruits and vegetables and less alcohol and tobacco in daily life; the larger the value of ADL, the stronger the ability of daily activities of older people, that is, the more independent; the value of medical expenditure The larger the value, the older people spend more on hospitalization and outpatient expenses; the larger the value of outdoor activities, the higher the frequency of outdoor activities; the larger the value of the place of residence variable, the farther away from the urban area

### Correlation analysis

When the independent variables in the model have strong multicollinearity, it will affect the reliability of the analysis results of the mediation effect model. Statistical methods for testing multicollinearity include the Pearson correlation test and variance inflation factor (VIF) test. In this paper, Pearson correlation test is used to test all variables in the model, and the test results are shown in Table 3.

**Table 3:** Pearson correlation analysis

Variables	ME	OA	HS	MH	CA	EH	ADL	PR	Age	Size	GDP
ME	1										
OA	-0.044***	1									
HS	-0.049***	0.183***	1								
MH	-0.040***	0.129***	0.421***	1							
CA	-0.009***	0.204***	0.121***	0.007	1						
EH	-0.028***	0.136***	0.085***	-0.041***	0.077***	1					
ADL	-0.050***	0.531***	0.268***	0.194	0.324***	-0.001	1				
PR	-0.064***	0.030***	-0.031***	-0.032***	-0.133***	-0.100***	0.061***	1			
Age	-0.019**	-0.372***	-0.070***	-0.056***	-0.233***	-0.038***	-0.625***	0.001	1		
Size	-0.019**	-0.053***	0.002	0.010	0.039***	0.007	-0.058***	0.095***	0.054***	1	
GDP	0.120***	-0.050***	0.018**	0.033***	0.003	0.047***	-0.070***	-0.325***	-0.008	-0.135***	1

Among them, *ME* refers to medical expenditure, *OA* refers to outdoor activities, *HS* refers to health status, *CA* refers to cognitive ability, *EH* refers to eating habits, *ADL* refers to daily activities, *PR* refers to a place of residence, *GDP* refers to per capita gross product. We are interested in the correlation coefficient and significance in the table, but since the correlation coefficient between some two variables does not appear in the results, the absolute value of the coefficient is greater than 0.8 under the premise that it is significant, so it is not necessary to proceed with the variance expansion coefficient Inspection (VIF)

Significant level: *p < 0.10; **p < 0.05; ***p < 0.01

It can be seen from Table 3 that the absolute value of the correlation coefficient between variables is at most 0.625. Statistically, it is generally believed that serious multicollinearity may occur only when the correlation coefficient between independent variables exceeds 0.8. Therefore, the model constructed in this paper does not have serious multicollinearity problems among variables, and the model regression analysis can be carried out in the next step.

### Mediating effect regression analysis

#### Mediating effect of mental health

To minimize bias, we controlled for time and province-fixed effects in all regressions. Our analysis focuses on the relationship between outdoor activities and medical expenditure, with mental health and health status as intermediary variables. The results, presented in Table 4, indicate that increased outdoor activities among older people significantly reduce their medical expenditure when mental health and health status are not considered. Furthermore, we observe a positive impact of outdoor activities on mental health, supporting hypothesis H1a. Model 6 demonstrates that improved mental health significantly enhances overall health status among older people, confirming hypothesis H3a. Additionally, hypothesis H4 is verified, as increased outdoor activities significantly improve general health status. In Model 7, incorporating all mediator and control variables, we find that all mediator variables have a significant negative impact on medical expenditure, providing evidence for hypotheses H6 and H5. Overall, the regression results validate the theoretical hypothesis that the mental health of older people serve as an important channel through which outdoor activities affect medical expenditure.

**Table 4:** Regression results of outdoor activities, mental health, health status and medical expenditure

Variables	Model 1(a)	Model 2	Model 6	Model 7
	**ME**	**MH**	**HS**	**ME**
OA	-500.519*** (-2.64)	0.276*** (3.95)	0.014*** (3.02)	-457.461** (-2.43)
HS	–-	–-	–-	-906.681** (-3.24)
MH	–-	–-	0.002*** (6.27)	-86.626*** (-4.31)
CA	-50.290*** (-4.03)	-0.029*** (-5.49)	0.025*** (46.98)	-51.432*** (-4.12)
EH	-742.418*** (-3.99)	-0.325*** (-4.46)	0.067*** (13.40)	-717.147*** (-3.81)
ADL	-79.812*** (-4.32)	0.166*** (24.26)	0.011*** (24.96)	-51.455*** (-2.64)
PR	-868.180** (-2.31)	-0.888*** (-6.59)	-0.039*** (-4.13)	-1000.35*** (-2.66)
Age	-184.433*** (-6.85)	0.118*** (11.79)	0.010*** (14.82)	-162.494*** (-6.03)
Size	-144.892 (-1.13)	0.477*** (10.11)	0.012*** (3.57)	-82.440 (-0.64)
GDP	0.969*** (3.94)	0.001*** (7.30)	-0.000*** (-2.38)	1.047*** (4.22)
Constant	-100,153.1*** (-2.63)	-82.690*** (-4.57)	3.066*** (2.63)	-106,430.9*** (-2.78)
Observation	15,874	15,874	15,874	15,874
Year FE	Yes	Yes	Yes	Yes
Province FE	Yes	Yes	Yes	Yes

Model 1(a) represents the total effect of outdoor activities on the medical expenditure of older people without the participation of the two variables of health status and mental health. It is obtained according to Eq. (1). The difference is that the model combines cognitive ability, diet Habits and activities of daily living served as control variables. Model 2, Model 6, and Model 7 all correspond to Eq. 2, Eq. 6, and Eq. 7

Robust standard errors in brackets, significant level: *p < 0.10; **p < 0.05; ***p < 0.01

#### Mediating effect of cognitive ability

We examine how the cognitive ability of older people mediate the relationship between outdoor activities and medical expenditure. The regression results are presented in Table 5. Table 5 demonstrates the impact of outdoor activities on medical expenditures, excluding the influence of cognitive abilities. The findings align with those from the previous section, showing a significant negative relationship, confirming that outdoor activities have a direct effect on reducing medical expenditure. Model 3 reveals a positive relationship between outdoor activities and cognitive ability, indicating that increased participation in outdoor activities improves cognitive ability, supporting hypothesis H1b. Model 6 indicates that improved cognitive ability significantly enhances the overall health status of older people, validating hypothesis H3a. Lastly, by examining Model 7, we observe the direct impact of cognitive ability on medical expenditure. The regression results reveal that as cognitive ability improves, medical expenditure significantly decreases, supporting hypothesis H2b.

**Table 5:** Regression results of outdoor activities, cognitive ability, health status and medical expenditure

Variables	Model 1(b)	Model 3	Model 6	Model 7
	**ME**	**CA**	**HS**	**ME**
OA	-498.114*** (-2.63)	0.495*** (4.77)	0.014*** (3.02)	-457.461** (-2.43)
HS	–-	–-	–-	-906.681** (-3.24)
CA	–-	–-	0.025*** (46.98)	-51.432*** (-4.12)
MH	-106.291*** (-5.55)	-0.063*** (-5.46)	0.002*** (6.27)	-86.626*** (-4.31)
EH	-829.153*** (-4.50)	0.960*** (8.89)	0.067*** (13.40)	-717.147*** (-3.81)
ADL	-80.232*** (-4.36)	0.353*** (34.83)	0.011*** (24.96)	-51.455*** (-2.64)
PR	-910.988** (-2.43)	-1.019*** (-5.63)	-0.039*** (-4.13)	-1000.35*** (-2.66)
Age	-167.475*** (-6.20)	-0.067*** (-4.45)	0.010*** (14.82)	-162.494*** (-6.03)
Size	-108.127 (-0.84)	0.288*** (4.14)	0.012*** (3.57)	-82.440 (-0.64)
GDP	1.042*** (4.20)	0.000 (1.59)	-0.000*** (-2.38)	1.047*** (4.22)
Constant	-108,823.8*** (-2.84)	-7.683 (-0.21)	3.066*** (2.63)	-106,430.9*** (-2.78)
Observation	15,874	15,874	15,874	15,874
Year FE	Yes	Yes	Yes	Yes
Province FE	Yes	Yes	Yes	Yes

Model 1(a) represents the total effect of outdoor activities on the medical expenditure of older people without the participation of the two variables of health status and cognitive ability. It is obtained according to Eq. (1). The difference is that the model combines mental health, diet Habits and activities of daily living served as control variables. Model 3, Model 6, and Model 7 all correspond to Eq. 3, Eq. 6, and Eq. 7

Robust standard errors in brackets, significant level: *p < 0.10; **p < 0.05; ***p < 0.01

#### Mediating effect of eating habits

Table 6 demonstrates the mediating effect of older adults eating habits in examining the influence of outdoor activities on their medical expenditure. Initially, regression results reveal a significant negative impact of outdoor activities on medical expenditure, supporting the credibility of the conclusions on medical spending. Model 4 highlights that increased outdoor activity frequency positively affects older adults eating habits, leading to the development of healthier eating habits and the abandonment of poor ones, validating hypothesis H1c. Furthermore, Model 6 indicates that improved eating habits positively impact the overall health status of older people, verifying hypothesis H3c. Lastly, Model 7 confirms the significantly negative impact of eating habits on medical expenditures, indicating that good eating habits play a crucial role in reducing medical costs and verifying hypothesis H2c.

**Table 6:** Regression results of outdoor activities, eating habits, health status and medical expenditure

Variables	Model 1(c)	Model 4	Model 6	Model 7
	**ME**	**EH**	**HS**	**ME**
OA	-590.641*** (-3.23)	0.153*** (20.94)	0.014*** (3.02)	-457.461** (-2.43)
HS	–-	–-	–-	-906.681** (-3.24)
EH	–-	–-	0.067*** (13.40)	-717.147*** (-3.81)
CA	-57.444*** (-4.62)	0.005*** (8.89)	0.002*** (6.27)	-51.432*** (-4.12)
MH	-106.711*** (-5.54)	-0.004*** (-4.46)	0.025*** (46.98)	-86.626*** (-4.31)
ADL	-54.542*** (-2.89)	-0.008*** (-12.07)	0.011*** (24.96)	-51.455*** (-2.64)
PR	-866.172** (-2.30)	-0.127*** (-8.74)	-0.039*** (-4.13)	-1000.35*** (-2.66)
Age	-168.286*** (-6.25)	-0.003*** (-3.31)	0.010*** (14.82)	-162.494*** (-6.03)
Size	-103.486 (-0.80)	0.013*** (2.64)	0.012*** (3.57)	-82.440 (-0.64)
GDP	1.063*** (4.28)	0.000 (-0.02)	-0.000*** (-2.38)	1.047*** (4.22)
Constant	-112,003.9*** (-2.92)	3.560* (1.84)	3.066*** (2.63)	-106,430.9*** (-2.78)
Observation	15,874	15,874	15,874	15,874
Year FE	Yes	Yes	Yes	Yes
Province FE	Yes	Yes	Yes	Yes

Model 1(a) represents the total effect of outdoor activities on the medical expenditure of older people without the participation of the two variables of health status and eating habits. It is obtained according to Eq. (1). The difference is that the model combines mental health, cognitive Ability and activities of daily living were used as control variables. Model 4, Model 6, and Model 7 all correspond to Eq. 4, Eq. 6, and Eq. 7

Robust standard errors in brackets, significant level: *p < 0.10; **p < 0.05; ***p < 0.01

#### Mediating effect of daily activities ability

This study investigates the mediating effect of older adults' daily activity performance on the relationship between outdoor activities and medical expenditures. Table 7 provides a summary of the findings. The first column reports the overall effect of outdoor activities on medical expenditure, revealing a significant negative impact. Model 5 demonstrates that increased frequency of outdoor activities positively affects daily activity performance among older people, confirming hypothesis H1d. Model 6 shows that improved daily activity performance contributes to better overall health, verifying hypothesis H3d. Lastly, the direct impact of daily activity performance on medical expenditures indicates that improvement in daily activities helps reduce medical expenses, supporting hypothesis H2d.

**Table 7:** Regression results of outdoor activities, ADL, health status and medical expenditure

Variables	Model 1(d)	Model 5	Model 6	Model 7
	**ME**	**ADL**	**HS**	**ME**
OA	-707.478*** (-4.02)	3.845*** (46.88)	0.014*** (3.02)	-457.461** (-2.43)
HS	–-	–-	–-	-906.681** (-3.24)
ADL	–-	–-	0.011*** (24.96)	-51.455*** (-2.64)
CA	-66.909*** (-5.55)	0.218*** (32.71)	0.025*** (46.98)	-51.432*** (-4.12)
EH	-714.937*** (-3.92)	-1.024*** (-12.09)	0.067*** (13.40)	-717.147*** (-3.81)
MH	-123.597*** (-6.70)	0.227*** (24.64)	0.002*** (6.27)	-86.626*** (-4.31)
PR	-999.561*** (-2.65)	0.555*** (3.61)	-0.039*** (-4.13)	-1000.35*** (-2.66)
Age	-124.725*** (-5.92)	-0.755*** (-74.49)	0.010*** (14.82)	-162.494*** (-6.03)
Size	-65.437 (-0.51)	-0.444*** (-7.68)	0.012*** (3.57)	-82.440 (-0.64)
GDP	1.051*** (4.23)	0.000 (1.16)	-0.000*** (-2.38)	1.047*** (4.22)
Constant	-112,184.3*** (-2.93)	48.088** (2.04)	3.066*** (2.63)	-106,430.9*** (-2.78)
Observation	15,874	15,874	15,874	15,874
Year FE	Yes	Yes	Yes	Yes
Province FE	Yes	Yes	Yes	Yes

Model 1(a) represents the total effect of outdoor activities on the medical expenditure of older people without the participation of the two variables of health status and daily activity ability. It is obtained according to Eq. (1). The difference is that the model combines mental health, cognitive ability and eating habits were used as control variables. Model 5, Model 6, and Model 7 all correspond to Eq. 5, Eq. 6, and Eq. 7

Robust standard errors in brackets, significant level: *p < 0.10; **p < 0.05; ***p < 0.01

### Mediation path and effect test

**Fig. 3:**
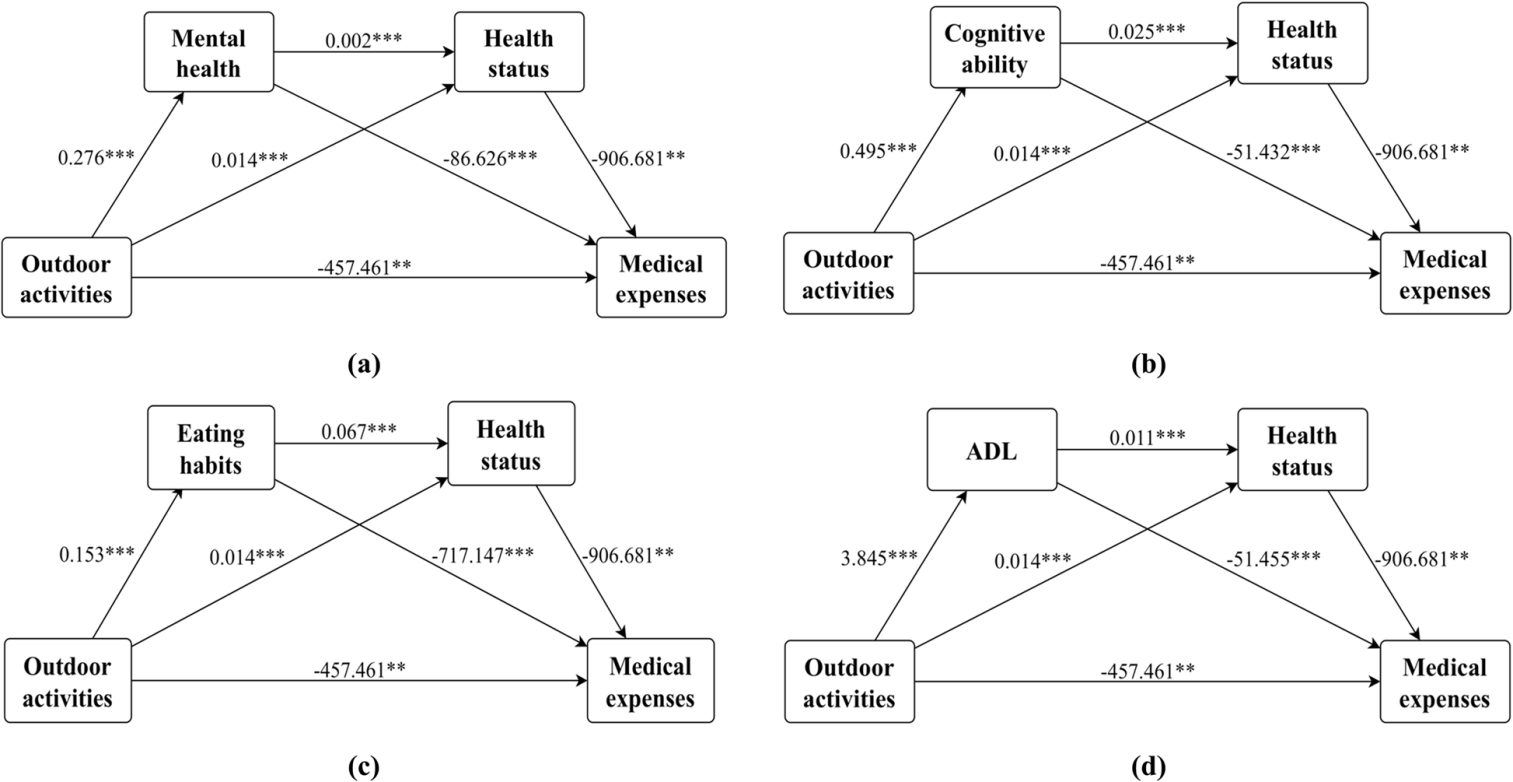
Coefficients and significance of transduction pathways. Note: The figure is based on the clear impact path constructed by the results of the regression model, in which (**a**) indicates that the mental health and overall health of older people are used as mediating variables to establish a double-chain mediation effect; **b** indicates that older people Cognitive ability and health status are the mediating variables to establish a double-chain mediation effect; **c** indicates the establishment of a double-chain mediation effect when older people’s eating habits and health status are mediating variables; **d** indicates older people’s daily life ability and health status as the mediating variable to establish a double-chain mediating effect. In addition, significant levels: *p < 0.10; **p < 0.05; ***p < 0.01

In the theoretical review, we identify the mediating variables involved in the influence of outdoor activities on medical expenditure among older people. Drawing from previous studies, we develop a preliminary plan for the influence pathway. By employing techniques similar to Baron and Kenny (1986) [27], we systematically validate our path planning and theoretical framework through the construction of multiple models. The regression results of these models are presented in the previous section. To demonstrate the strength and direction of the variable relationship, as well as the causal link between observed and latent variables, we employ a flow chart displaying correlation coefficients and their significance levels (Fig. 3). Furthermore, we utilize Bootstrap's method (the reps are 1000) to estimate the significance of each path coefficient and present the results, including the effects and significance of specific action paths, in Table 8.

**Table 8:** Mediation effects testing

Relationship	Coefficient	Z-value	Bootstrap CI (95%)	Ratio of total effect
**Mediating effect (a): Mental health**				
OA →ME	-458.525	-2.48**	[-766.8171, -63.2585]	91.31%
OA →MH →ME	-24.187	-3.07***	[-43.8521, -12.0836]	4.82%
OA →HS →ME	-13.071	-2.10**	[-29.5646, -5.1364]	2.60%
OA →MH →HS →ME	-6.401	-2.59**	[-12.9381, -2.7091]	1.27%
**Mediating effect (b): Cognitive ability**				
OA →ME	-458.525	-2.48**	[-766.8171, -63.2585]	92.05%
OA →CA →ME	-25.517	-3.23***	[-47.2384, -13.5047]	5.12%
OA →HS →ME	-13.071	-2.10**	[-29.5646, -5.1364]	2.62%
OA →CA →HS →ME	-0.999	-2.52**	[-2.0607, -0.4611]	0.20%
**Mediating effect (c): Eating habits**				
OA →ME	-458.525	-2.48**	[-766.8171, -63.2585]	78.84%
OA →EH →ME	-109.707	-3.51***	[-167.4904, -52.9404]	18.86%
OA →HS →ME	-13.071	-2.10**	[-29.5646, -5.1364]	2.25%
OA →EH →HS →ME	-0.308	-2.74**	[-0.5258, -0.1358]	0.05%
**Mediating effect (d): Activities of daily living**				
OA →ME	-458.525	-2.48**	[-766.8171, -63.2585]	65.51%
OA →ADL →ME	-190.583	-2.70***	[-344.5745, -61.6495]	27.23%
OA →HS →ME	-13.071	-2.10**	[-29.5646, -5.1364]	1.87%
OA →ADL →HS →ME	-37.722	-3.32***	[-59.4565, -16.2747]	5.39%

The Z-value is a robust estimate based on the Sobel test [28]

The ratio of the total effect is the corresponding coefficient value divided by the sum of all coefficient values (total effect)

Significant level: *p < 0.10; **p < 0.05; ***p < 0.01

### Robustness check

Through the specific analysis and simple verification of the regression results above, we will next test the robustness of the regression results. We replaced the medical expenditure of older people with hospitalization expenses and outpatient expenses and replaced the health status of older people with the investigator's objective health evaluation of older people. And regress the models in Tables 4, 5, 6, and7. The regression results are shown in Table 9. From the results, it can be seen that the significance of these mediation effects is strong or weak, but overall, they still exist. Therefore, we have reason to believe that the selection of these mediation variables in this paper is robust.

**Table 9:** Robustness test: Replace the explained variable

Variables	Model 1	Model 2	Model 3	Model 4	Model 5	Model 6	Model 7	Model 8
	IE	Mediation	HS	IE	OE	OE	HSE	ME
OA	–-	–-	0.014*** (3.02)	-179.257 (-1.61)	–-	-279.268*** (-2.70)	0.014*** (3.93)	-456.971** (-2.42)
HS (HSE)	–-	–-	–-	-344.591** (-2.04)	–-	-564.173*** (-3.47)		-1073.107** (-2.57)
MH	–-	–-	0.025*** (46.98)	-33.174*** (-2.74)	–-	-53.547*** (-4.63)	0.012*** (31.48)	-95.728*** (-4.60)
OA|MH	-195.893* (-1.76)	0.278*** (4.00)	–-	–-	-306.292*** (-2.95)	–-		
CA	–-	–-	0.002*** (6.27)	-35.548*** (-4.59)	–-	-15.943** (-2.35)	0.002*** (6.47)	-51.557*** (-4.11)
OA|CA	-202.209* (-1.81)	0.495*** (4.77)	–-	–-	-295.904*** (-2.86)	–-		
EH	–-	–-	0.067*** (13.40)	-458.436*** (-4.12)	–-	-258.342** (-2.41)	0.014*** (3.90)	-762.311*** (-4.12)
OA|EH	-257.921** (-2.41)	0.153*** (20.94)			-332.721*** (-3.28)			
ADL	–-	–-	0.011*** (24.96)	-35.471*** (-3.17)	–-	-15.727 (-1.50)	0.018*** (50.85)	-41.044** (-2.01)
OA|ADL	-335.413*** (-3.28)	3.845*** (46.88)	–-	–-	-372.065*** (-3.78)	–-		
Constant	–-	–-	3.066*** (2.63)	-42,441.84* (-1.82)	–-	-64,006.19*** (-2.76)	3.140*** (3.50)	3.140*** (3.50)

Model 1 represents the influence of explanatory variables on the explained variables in the context of different intermediary variables, where "OA | MH" represents the effect of OA on the explained variables in the context of MH influence coefficient. For the pertinence of the discussion, we do not report the constant term coefficients for Model 1. The above settings are also applicable in model 2 and model 5, the only difference is that the explained variable is different; in addition, we canceled the report of the number of observations, fixed effects and control variables, because they are the same as the regression above. Model 7 and Model 8 will replace the health status of older people with the investigator's evaluation of the health status of older people (HSE)

*IE* represents inpatient expenses, *OE* represents outpatient expenses

Robust standard errors in brackets, significant level: *p < 0.10; **p < 0.05; ***p < 0.01

We have four parallel variables (ME, CA, EH, ADL). In our identification strategy, when one of the variables is used as the explained variable, the other three variables will be added to the regression equation as explanatory variables. This is a typical system of simultaneous equations, that is, there is an inherent relationship between the variables in different equations, and the explanatory variable of one equation is the explained variable of another equation. However, we only show the main results, and default to the independence of the four parallel variables, which is similar to the "seemingly unrelated regression" (SUR) in a multi-equation system, that is, there is no intrinsic relationship between the equations, but the relationship between each equation is There is a correlation between the disturbance terms. Therefore, the SUR method was used to verify the correlation between the equations of the four variables. The results are shown in Table 10. Panel A reports the estimation results of OLS and the estimation results of SUR. The regression results of the two show a high degree of consistency. Panel B reports the correlation analysis results of SUR. Even though the BP test results reject the null hypothesis that the disturbance terms of each equation are independent of each other at the 1% significance level, the correlation between the equations is low (the highest is 0.25), so SUR does not provide better regression results than OLS. On the premise of ensuring that the OLS estimation is valid, we will continue to promote robustness testing.

**Table 10:** Robustness test: seemingly unrelated regression estimation

Variables	MH		CA		EH		ADL	
	OLS	SUR	OLS	SUR	OLS	SUR	OLS	SUR
Panel A								
OA	0.997*** (15.23)	0.997*** (15.24)	1.964*** (17.47)	1.964*** (17.47)	0.118*** (17.48)	0.118*** (17.48)	4.347*** (56.24)	4.347*** (56.25)
PR	-0.420*** (-3.30)	-0.420*** (-3.30)	-4.223*** (-19.32)	-4.223*** (-19.33)	-0.157*** (-11.98)	-0.157*** (-11.98)	0.948*** (6.31)	0.948*** (6.31)
Age	-0.009 (-1.00)	-0.009 (-1.00)	-0.338*** (-22.57)	-0.338*** (-22.57)	0.00162* (1.79)	0.00162* (1.79)	-0.842*** (-81.71)	-0.842*** (-81.73)
Size	0.149*** (3.02)	0.149*** (3.02)	0.737*** (8.68)	0.737*** (8.68)	0.017*** (3.38)	0.017*** (3.38)	-0.238*** (-4.08)	-0.238*** (-4.08)
GDP	0.000*** (3.97)	0.000*** (3.97)	-0.000*** (-4.09)	-0.000*** (-4.09)	0.000*** (3.05)	0.000*** (3.05)	-0.000*** (-8.11)	-0.000*** (-8.11)
Constant	53.55*** (55.04)	53.55*** (55.05)	112.6*** (67.38)	112.6*** (67.39)	2.626*** (26.12)	2.626*** (26.13)	108.2*** (94.19)	108.2*** (94.21)
Observation	15,874	15,874	15,874	15,874	15,874	15,874	15,874	15,874
Panel B (Breusch–Pagan test of independence: chi2(6) = 1795.068, Pr = 0.0000)								
MH	1.000		–-		–-		–-	
CA	-0.011		1.000		–-		–-	
EH	-0.047		0.057		1.000		–-	
ADL	0.211		0.250		-0.022		1.000	

The explained variables are mental health, cognitive ability, eating habits and ADL. In this article, these four variables are all parallel mediating variables. Panel A shows the regression results of outdoor activities on the explained variables, including the OLS method and the SUR method. Panel B shows the correlation coefficient matrix between the four explained variables and the test results of "no contemporaneous correlation" between the disturbance terms of each equation when using the SUR method for regression estimation

Robust standard errors in brackets, significant level: *p < 0.10; **p < 0.05; ***p < 0.01

To verify the impact of outdoor activities on medical expenditure, we employ instrumental variables to test the regression results. Our statistics on the frequency of outdoor activities for older people are based on low-intensity physical activities like Tai Chi, square dancing, and outdoor socializing. These activities are commonly organized within communities in China. By using the frequency of community organization activities as an instrumental variable, we tested the robustness of the regression results. The results, as shown in Table 11 [29], the Kleibergen-P F statistics are all greater than 10, which significantly rejects the original hypothesis of "under-identification of cognitive variables". The Cragg-D F statistics are all greater than 10, which is significantly greater than the critical value of the Stock-Yogo weak identification test at the 10% level. This shows that the selection of instrumental variables is reasonable and efficient, indicate that outdoor activities still significantly affect the medical expenditure of older people and most intermediary variables, confirming the direction of the effect and verifying robustness [30].

**Table 11:** Robustness test: Instrumental variable method

Variables	Model 1	Model 2	Model 3	Model 4	Model 5	Model 6	Model 7
	ME	MH	CA	EH	ADL	HS	ME
OA	–-	18.250*** (5.31)	11.013*** (3.18)	-2.030*** (-3.31)	7.474*** (4.90)	0.436*** (3.20)	-13,959.05*** (-2.80)
HS	–-	–-	–-	–-	–-	–-	-119.418 (-0.27)
MH	–-	–-	-0.167*** (-7.13)	0.005*** (1.47)	0.157*** (6.19)	0.025*** (25.90)	-8.556 (-0.30)
OA|MH	-14,221.9*** (-3.18)	–-	–-			–-	–-
CA	–-	-0.073*** (-5.95)	–-	0.009*** (4.54)	0.121*** (8.03)	0.001*** (2.68)	28.046* (1.86)
OA|CA	-13,703.93*** (-2.89)	–-	–-	–-		–-	–-
EH	–-	-3.439*** (-5.73)	-0.907*** (-1.47)	–-	-1.506*** (-5.90)	-0.005 (-0.23)	1460.778* (1.74)
OA|EH	-16,978.62*** (-2.43)	–-		–-	–-	–-	–-
ADL	–-	-0.515*** (-3.93)	-0.059*** (-0.45)	0.071*** (3.19)	–-	-0.005 (-0.96)	414.123** (2.24)
OA|ADL	-10,911.32*** (-3.27)	–-	–-	–-	–-	–-	–-
Constant	–-	11.683* (1.79)	62.701*** (9.98)	7.902*** (5.97)	63.546*** (7.76)	-0.578** (-2.56)	49,296.41*** (5.97)
Cragg-D F	–-	33.227	28.651	14.647	44.569	27.352	26.211
Kleibergen-P F	–-	33.919	29.129	14.971	45.532	27.837	26.700

Model 1 indicates that in the context of a mediating effect, the instrumental variable method is used to explore the direct impact of outdoor activities on medical expenditures, where "OA|MH" is the direct impact of outdoor activities on medical expenditures in the context of mental health. Models 2–6 show the impact of outdoor activities on various mediator variables. In this table, we do not report common control variables, but report constant terms (except model 1) and correlation tests of instrumental variables (both Cragg-D F and Kleibergen-P F are Weak identification tests)

Robust standard errors in brackets, significant level: *p < 0.10; **p < 0.05; ***p < 0.01

### Further research

The factors driving outdoor activities among older adults vary based on their living environments, impacting their medical expenses. Urban areas receive more government investment in infrastructure, creating a noticeable gap in infrastructure and lifestyle between urban and rural areas. Urban older adults have greater access to a wider range of outdoor activities due to diverse options and better infrastructure. As a result, they show more interest and participate more frequently in outdoor activities compared to their rural counterparts. To account for these differences, we classified older adults into urban and rural groups, with 8,780 urban older adults (places of residence are marked 1 and 2) and 7,094 rural older adults (places of residence are marked 3). We compared Tables 4, 5, 6, and7 to observe the types of activities and the level of infrastructure based on their place of residence. Then, we conducted group regression to examine the direct and indirect effects of outdoor activities on medical expenditure for different groups of older adults. The results are presented in Table 12.

**Table 12:** Subsample regression of older people living in different regions

Variables	Urban group				Rural group			
	ME	Mediation	HS	ME	ME	Mediation	HS	ME
OA	–-	–-	0.011** (1.98)	-678.191*** (-2.65)	–-	–-	0.017*** (2.55)	-180.78 (-0.64)
HS	–-	–-	–-	-797.992** (-2.06)	–-	–-	–-	-994.357** (-2.49)
MH	–-	–-	0.025*** (35.98)	-112.059*** (-4.22)	–-	–-	0.024*** (30.11)	-48.852 (-1.61)
OA|MH	-728.292*** (-2.84)	0.307*** (3.23)	–-	–-	-214.449 (-0.76)	0.221*** (2.17)	–-	–-
CA	–-	–-	0.002*** (4.59)	-54.569*** (-2.86)	–-	–-	0.002*** (4.53)	-42.149** (-2.55)
OA|CA	-721.001*** (-2.80)	0.592*** (4.53)	–-	–-	-212.231 (-0.76)	0.315* (1.92)	–-	–-
EH	–-	–-	0.053*** (7.87)	-519.858* (-1.93)	–-	–-	0.084*** (11.51)	-896.013*** (-3.48)
OA|EH	-771.382*** (-3.07)	0.148*** (15.00)	–-	–-	-352.160 (-1.31)	0.157*** (14.49)	–-	–-
ADL	–-	–-	0.010*** (16.93)	-68.434*** (-2.60)	–-	–-	0.012*** (18.91)	-27.334 (-0.95)
OA|ADL	-984.253*** (-4.18)	3.863*** (34.11)	–-	–-	-345.984 (-1.29)	3.742*** (31.42)	–-	–-
Constant	–-	–-	6.655*** (3.60)	-46,030.76 (-0.83)	–-	–-	1.625 (0.95)	-190,272.6*** (-2.66)

In the table, 1–4 are samples of older people in the urban group, and columns 5–8 are samples of older people in the rural group; columns 1 and 5 indicate the total impact of outdoor activities on medical expenditure in the context of studying the corresponding intermediary variables, where "OA|MH" indicates the total effect of OA on ME when the MH variable is not added, and the same is true for other items; the second and sixth columns indicate the impact of outdoor activities on the mediator variable, where "OA|MH" Indicates the impact of OA on MH; columns 3 and 7 indicate the impact of outdoor activities and various intermediary variables on the overall health of older people; columns 4 and 8 indicate the impact of outdoor activities and various intermediary variables on the medical expenditure of older people. The sample sizes of the urban and rural groups are 8780 and 7094

Robust standard errors in brackets, significant level: *p < 0.10; **p < 0.05; ***p < 0.01

From Table 12, it is observed that the impact of outdoor activities on medical expenditure differs for older adults in rural areas compared to the total sample. When other mediator variables are controlled, the frequency of outdoor activities no longer has a significant effect on medical expenditure for rural older adults. This suggests that outdoor activities in rural areas do not directly influence medical expenditure. However, it was found that outdoor activities have a significant impact on the overall health status of rural older adults, as well as on various intermediary variables such as mental health, cognitive ability, eating habits, and daily activities. This indicates that there is a significant path from outdoor activities to mediator variables to health status for rural older adults. Nevertheless, the health benefits resulting from this path are not directly reflected in medical expenditure. Furthermore, it was observed that the path from mediator variables to health status to medical expenditure is significant for the entire older adults’ sample. Specifically, for rural older adults, although the estimated coefficient of outdoor activities on medical expenditures is negative (-180.78), it has lost any statistical significance, that is, these mediator variables have a masking effect rather than a mediating effect. Controlling for these variables significantly amplifies the differences in medical spending between rural and urban older adults’ populations.

## Discussion

Our study examines the impact of outdoor activities among older adults on healthcare expenditures, as well as the potential mechanisms underlying this impact. Utilizing cross-sectional data from a survey of elderly individuals in China, we investigate the causal relationships and pathways of these variables. We find that amidst the backdrop of population aging, increased frequency of outdoor activities among older adults contributes to a reduction in healthcare expenditures. In this process, improvements in the psychological well-being, cognitive abilities, dietary habits, and daily activity levels of older adults serve as facilitating pathways. While our study may appear to address a novel topic, it is underpinned by a substantial body of existing research to establish its connectivity. Below, we provide a detailed discussion of the academic positioning, practical significance, limitations, and avenues for future research of this study.

### Content integration

The academic positioning of this study is that of an integrative research endeavor with connective attributes. The juxtaposition of outdoor activities and healthcare expenditures may initially seem inherently humorous — is there truly a cost-saving aspect to outdoor pursuits? However, behind this seemingly theatrical conclusion lies a robust theoretical foundation. In economics, the demonstration of a causal relationship may initially be met with skepticism, but as the mechanisms underlying it are unveiled, initial incredulity often transforms into enlightenment. Returning to the topic at hand, we have identified numerous similar pathways based on existing research. As articulated in Literature review and research hypothesis Section, a plethora of studies have concentrated on the left and right sides of these pathways, yet research that bridges the gap between both ends of the spectrum remains unpublished [31]. This constitutes the primary focus of our work — to connect the outdoor activities of older adults with healthcare expenditures through existing intermediary mechanisms, thereby completing the causal chain.

In the process of connecting these pathways, our study does not conflict with existing research; instead, it reinforces their findings. During the Covid-19 pandemic, there has been an increase in the prevalence of mental health issues. In England, one-fifth of adults experienced some form of depression in the first quarter of 2021 — more than twice the rate observed before the pandemic [32]. Similar research on the impact of major public health events leading to increased social distancing and subsequent effects on psychological well-being abounds [33]. While external factors contribute to increased social distancing, especially during events such as pandemics, older adults experience it more due to internal factors associated with aging, such as declining physical function. Whether driven by internal or external factors, the logic underlying these studies is analogous, leading to convergent conclusions [34] [35]. Toshiro Suzuki (2010) assessed the behavior of 53 elderly individuals living alone using infrared sensors and found that those who spent little time outdoors often exhibited cognitive decline [36]. Louise-Ann Leyland (2019) conducted outdoor activity interventions with 100 elderly individuals, with results indicating significant improvements in psychological well-being and cognitive abilities through outdoor cycling interventions [37]. Mika Kimura (2013) demonstrated through a cluster-randomized trial that outdoor activities among older adults improved their dietary habits [38]. Linda Burke (2010) similarly showed the positive effects of appropriate physical activity on dietary intake among older adults [39]. Miriam Cabrita (2017) indicated in her study that compared to indoor activities, elderly participation in outdoor activities was more closely associated with increased levels of daily activity [40]. These studies all demonstrate conclusions similar to those of our study: the beneficial health effects of increased outdoor activity frequency among older adults are evident. However, the specificity of the research topics confines them to the left side of the pathway, whereas our integrative study allows us to consider both sides of the pathway. The health improvements brought about by outdoor activities should directly translate into health benefits for older adults, namely a reduction in healthcare expenditures.

X. Sun (2020) collected panel data from 2,938 elderly individuals in rural China and estimated the impact of depressive symptoms and depression on healthcare expenditures using two-part and four-part models. Their results indicate that psychological health status significantly affects individual medical expenses, accounting for a cumulative total of 47.26% of expected personal healthcare expenses [41]. Prior to this, Benjamin G. Druss (1999) first estimated the relationship between depressive symptoms in older adults and health costs, with their research indicating that the most severely affected patients with depressive symptoms incurred healthcare costs 50% higher than those with milder symptoms [42]. Sungje Moon (2021) stated in their study that compared to elderly individuals without dementia, those with dementia incur higher lifetime healthcare costs [43]. Amy S. Kelley's (2015) findings reveal that dementia patients have an average out-of-pocket expenditure 81% higher than non-dementia patients, and the average total costs per dementia decedent significantly exceed those of individuals who died from heart disease, cancer, or other causes [44]. Yuan-Ting Lo (2016) conducted a dietary survey on 1,781 elderly individuals in Taiwan, with results indicating that the intake of fresh fruits and vegetables among older adults contributes to a reduction in all-cause mortality [45]. Roland Sturm's (2002) survey data show that hospitalization and outpatient costs for smokers and heavy drinkers are 21% higher than those for others, with medication expenditures increasing by 28% [46]. Hai-Yu Jin (2020) surveyed 5,300 elderly individuals in China, revealing that elderly individuals with lower levels of daily activity are associated with higher healthcare expenditures; specifically, elderly individuals with lower levels of daily activity incur $60.60 more in outpatient expenses than those with higher levels of physical activity [47]. The performance of older adults in terms of psychological health, cognitive abilities, dietary habits, and daily activity directly influences their healthcare expenditures, a point our study likewise confirms. Moreover, we analyze the impacts on the left side of these pathways. While we acknowledge that these manifestations in older adults affect their healthcare costs, ultimately, these manifestations are merely objective evaluations by researchers of their trial samples and are insufficient to support effective predictions and interventions by families or society for any individual elderly person. These studies may well reflect a common social phenomenon, but they are limited in their ability to intervene or improve specific manifestations in individual elderly individuals. Conversely, studies on the left side of the pathway demonstrate superiority in this regard. Therefore, our study not only connects these staged studies in terms of content and structure but also incorporates their strengths and avoids their weaknesses in research characteristics.

### Trade-offs in public health

Now that we have demonstrated that older adults’ outdoor activity has a causal impact on health care spending, should we encourage and urge older adults to increase their frequency of outdoor activity? I think we should look at it dialectically.

From a public health prevention perspective, an increase in the frequency of outdoor activities among older adults often coincides with an elevated risk of falls, presenting a significant concern. In the United States, approximately one-third of community residents aged 65 and older experience falls annually, with approximately 10% resulting in severe injuries. These falls and resulting injuries may lead to disabilities, loss of independence, and fear of falling, underscoring the necessity for public health prevention programs to address the risk of falls leading to severe injury [48–50]. Research indicates that falls outdoors on hard surfaces are more likely to cause injuries. Therefore, older adults should avoid using hard surfaces whenever possible, such as recreational walking areas [51]. Jennifer L. Kelsey (2012) demonstrated in their observational findings that older adults are more likely to sustain severe injuries from outdoor falls in familiar environments, while falls indoors are more common in unfamiliar environments [52]. Therefore, incorporating warnings about increased risks in unfamiliar environments into public health messaging may prove beneficial.

In appropriately addressing the prevention of outdoor falls among older adults, it becomes imperative to further discuss the societal and economic effects stemming from encouraging elderly participation in outdoor activities, representing an "economic calculus" from the public health sector. Public health departments need to balance the relationship between healthcare expenditures and health benefits. While falls may increase healthcare costs, regular outdoor activities may mitigate long-term healthcare expenditures and generate positive impacts on a broader economic scale. Research findings by Michael D. Hurd (2013) estimate that in 2010, the prevalence of dementia among individuals aged 70 and above in the United States was 14.7%. The annual monetary cost per person due to dementia was calculated at $56,290 or $41,689. These individual costs suggest that the total monetary cost of dementia in 2010 ranged between $157 billion and $215 billion, with approximately $110 billion in expenses covered by Medicare [53]. Therefore, reducing the incidence of chronic diseases and medical interventions can lower healthcare costs and alleviate societal burdens. Additionally, promoting healthy aging may delay the need for long-term care and medical services among older adults, thus reducing pressure on public health resources. Hence, public sectors should consider devising policies and investment schemes to encourage elderly participation in outdoor activities while concurrently implementing fall prevention measures to maximize health benefits and minimize healthcare expenditures. Moreover, public sectors should prioritize the societal and economic value of outdoor activities, including fostering social integration, enhancing community cohesion, and improving the quality of life for older adults [54]. By comprehensively considering health, social, and economic factors, public sectors can formulate comprehensive policies to promote the health and well-being of older adults while ensuring the effective utilization and sustainable development of societal resources.

In further analysis, we have uncovered disparities in the impact of outdoor activities on healthcare expenditures among elderly individuals in urban and rural areas. In urban regions, outdoor activities moderate healthcare expenditures, whereas in rural areas, they obscure them. These disparities can be attributed to limited resources, divergent medical beliefs and behaviors, as well as lifestyle and environmental distinctions between urban and rural settings. Rural areas often lack sufficient medical resources, leading elderly individuals to rely more on traditional therapies or home care rather than professional medical services. Additionally, elderly individuals in rural areas may face labor and livelihood pressures, constraining their opportunities for outdoor activities. Therefore, public sectors must not overlook outdoor activities and healthcare expenditures among elderly individuals in relatively underdeveloped regions.

### Limitations and development

Despite employing a rigorous research framework, methodology, and data collection, future studies should address several limitations. Firstly, reliance on self-reported outdoor activities among elderly individuals may introduce subjectivity and potential inaccuracies. Moreover, study findings may be confined to specific samples, such as geographic regions, cultural backgrounds, or socioeconomic statuses, impacting external validity. Secondly, establishing causality in the mediation models presents challenges due to confounding effects of unmeasured variables. Additionally, selecting appropriate mediator variables is crucial yet subjective, potentially leading to variations in study outcomes based on researchers' preferences. Lastly, since our data is cross-sectional, future research should incorporate longitudinal group studies to account for potential time lags between outdoor activities among older adults and healthcare expenditures, while considering longitudinal factors over time.

## Data Availability

The datasets generated and/or analyzed during the current study are available in the [https://opendata.pku.edu.cn/dataset.xhtml?persistentId=doi:10.18170/DVN/WBO7LK&version=2.1] repository. Due to partial missingness in the original data, the exact dataset used and/or analyzed during the current study is available from the corresponding author [Ge Zhu/gzhu2037@gmail.com] on reasonable request.

## Appendix

### About the selection and calculation of some variables

This is a specific detail about the selection and calculation of some variables. It includes related sources and calculation methods for Mental health (MH), Cognitive ability (CA), Eating habits (EH) and Activities of daily living (ADL). Table 13 shows the specific expression and composition structure of the above variables in the questionnaire.

The main calculation idea of these variables is score standardization. The task at this stage is to score the different variables of these elderly people so that there are individual differences between these elderly people on the same variable. As shown in Panel A of Table 13, this is the survey content of the mental health module (depression scale) on the questionnaire. There are 10 questions in total, and each question has 5 options. The larger the number of the option, it means that older adults have no relevant psychological problems. The higher the score, the healthier the mental health. In the process of standardization, we assume that when the answers to these 10 questions are all "1", older adults person's score in this module is 0, and we think that older adults have serious psychological problems. When the answers to these 10 questions are When the answers are all "5", the old man's score in this module is 100, and the old man is considered mentally healthy. As shown in Panel B, this is the survey content of the cognitive ability module on the questionnaire. There are 7 questions in total, and each question has 4 options. The serial number of the option indicates different types of responses or answers of older adults (relaxing the restrictions on the cognition of older adults, and older adults’s answer of “don’t know” or refusal to answer is also considered as an answer option), we assume that when the answers to these 7 questions are all "1", the old man's score in this module is 0, we think that the old man has severe cognitive impairment. When the answers to these 7 questions are all " 4", the old man's score in this module is 100, and the old man's cognition is considered to be relatively unobstructed. As shown in Panel C, this is the survey content of the eating habits module on the questionnaire. There are 4 questions in total, and each question has 4 options. This module is slightly different from the previous two modules, because regular intake of fresh fruits and vegetables is a good eating habit, but regular intake of tobacco and alcohol is a bad one. eating habits, so we make positive adjustments to the options before starting to standardize. At this time, the larger the options for all questions, the closer older adults are to good eating habits. We assume that when these four questions are all "1", older adults have poor eating habits, and when all the options are "4", older adults have good eating habits. As shown in Panel D, this is the survey content of the daily activity ability module on the questionnaire. There are 14 questions in total, and each question has 3 options. The larger the option number, the less obstacles or limitations older adults have in their daily life abilities. During the standardization process, we assume that when the answers to these 14 questions are all "1", we think that older adults has almost no ability to perform daily activities. When the answers to these 14 questions are all "3", we think that older adults have a high ability to carry out daily activities.

The above are the definition standards and calculation methods of the four parallel variables. Of course, we have to remind you that due to the design of the questionnaire, all scales do not perfectly fit the standards of the corresponding international scales (for example, the contents of the internationally recognized depression scale and ADL scale do not match the content of this questionnaire) There are obvious differences in the content), so the scale questions in Table 13 cannot be used as a standard to evaluate the relevant characteristics of non-in-sample individuals. All relevant characteristics we obtain about individuals within the sample range can only be limited to this study, that is, comparisons can only be made within the sample range.

**Table 13:** Related scales in the questionnaire

Project	Question	Options
Panel A Mental health		
Depression Scale		
A-1	Do you get annoyed by the little things?	1-Always 2-Often 3-Sometimes 4-Rarely 5-Never
A-2	Is it difficult for you to concentrate when doing things now?	1-Always 2-Often 3-Sometimes 4-Rarely 5-Never
A-3	Are you feeling sad or depressed?	1-Always 2-Often 3-Sometimes 4-Rarely 5-Never
A-4	Do you find it harder to do things as you get older?	1-Always 2-Often 3-Sometimes 4-Rarely 5-Never
A-5	Are you full of hopeless for your future life?	1-Always 2-Often 3-Sometimes 4-Rarely 5-Never
A-6	Are you feeling nervous or scared?	1-Always 2-Often 3-Sometimes 4-Rarely 5-Never
A-7	Do you feel as happy as you did when you were young?	1-Always 2-Often 3-Sometimes 4-Rarely 5-Never
A-8	Are you feeling lonely?	1-Always 2-Often 3-Sometimes 4-Rarely 5-Never
A-9	Do you feel like you can't move on with your life?	1-Always 2-Often 3-Sometimes 4-Rarely 5-Never
A-10	Are you currently experiencing poor sleep quality?	1-Always 2-Often 3-Sometimes 4-Rarely 5-Never
Panel B Cognitive Ability		
Cognitive Function Scale		
B-1	What are the things people usually use to cut paper called?	1-Wrong 2-Don’t know 3-Reject 4-Correct
B-2	Do apples grow on trees or in the soil?	1-Wrong 2-Don’t know 3-Reject 4-Correct
B-3	Who is the chairman of China now?	1-Wrong 2-Don’t know 3-Reject 4-Correct
B-4	Recognize a part of the body pointed by the investigator	1-Wrong 2-Don’t know 3-Reject 4-Correct
B-5	What is a hammer usually used for?	1-Wrong 2-Don’t know 3-Reject 4-Correct
B-6	Where is the nearest store near you?	1-Wrong 2-Don’t know 3-Reject 4-Correct
B-7	Please point to the window first and then the door	1-Wrong 2-Don’t know 3-Reject 4-Correct
Panel C Eating habits		
Eating Habits Scale		
C-1	Do you often eat fresh fruit?	1-Rarely 2-Sometimes 3-Often 4-Every day
C-2	Do you eat fresh vegetables regularly?	1-Rarely 2-Sometimes 3-Often 4-Every day
C-3	Do you smoke regularly?	1-Every day 2-Often 3-Sometimes 4-Rarely
C-5	Do you drink alcohol regularly?	1-Every day 2-Often 3-Sometimes 4-Rarely
Panel D Activities of daily living		
ADL Scale		
D-1	Are you limited in your daily activities because of health problems?	1-Almost limited 2-Generally limited 3-No limit
D-2	Do you need help bathing?	1-Very necessary 2-Normally necessary 3-Not necessary
D-3	Do you need help getting dressed?	1-Very necessary 2-Normally necessary 3-Not necessary
D-4	Do you need help when going to the toilet to relieve yourself?	1-Very necessary 2-Normally necessary 3-Not necessary
D-5	Do you need help doing indoor activities?	1-Very necessary 2-Normally necessary 3-Not necessary
D-6	Do you need help eating?	1-Very necessary 2-Normally necessary 3-Not necessary
D-7	Do you have bowel and bladder control?	1-Cannot control 2-General control 3-Able to control
D-8	Can you visit your neighbor's house alone?	1-Cannot 2-Difficult 3-Can
D-9	Can you go out shopping alone?	1-Cannot 2-Difficult 3-Can
D-10	Can you cook alone if needed?	1-Cannot 2-Difficult 3-Can
D-11	Can you do the laundry on your own if needed?	1-Cannot 2-Difficult 3-Can
D-12	Can you walk 2 km continuously?	1-Cannot 2-Difficult 3-Can
D-13	Can you squat and stand up three times in a row?	1-Cannot 2-Difficult 3-Can
D-14	Can you travel alone on public transport?	1-Cannot 2-Difficult 3-Can
